# Hand pattern indicates prostate cancer risk

**DOI:** 10.1038/sj.bjc.6605986

**Published:** 2010-11-30

**Authors:** A A Rahman, A Lophatananon, S Stewart-Brown, D Harriss, J Anderson, T Parker, D Easton, Z Kote-Jarai, R Pocock, D Dearnaley, M Guy, L O'Brien, R A Wilkinson, A L Hall, E Sawyer, E Page, J-F Liu, The UK Genetic Prostate Cancer Study Collaborators, British Association of Urological Surgeons' Section of Oncology, R A Eeles, K Muir

**Affiliations:** 1 Division of Epidemiology and Public Health, University of Nottingham, Queens Medical Centre, Nottingham NG7 2UH, UK; 2 Health Sciences Research Institute, Warwick Medical School, Warwick University, Coventry CV4 7AL, UK; 3 Nottingham Urology Centre, Nottingham University Hospital NHS Trust, Nottingham NG5 1PB, UK; 4 Royal Hallamshire Hospital, Glossop Road, Sheffield S10 2JF, UK; 5 School of Biomedical sciences, University of Nottingham, Queens Medical Centre, Nottingham NG7 2UH, UK; 6 CR-UK Genetic Epidemiology Unit, Strangeways Research Laboratories, Worts Causeway, Cambridge CB1 8RN, UK; 7 The Institute of Cancer Research, 15 Cotswold Road, Sutton, Surrey SM2 5 NG, UK; 8 Royal Devon and Exeter NHS Foundation Trust, Barrack Road, Exeter EX2 5DW, UK; 9 The Royal Marsden NHS Foundation Trust, Downs Road, Sutton SM2 5PT, UK; 10 Children's Brain Tumour Research, Division of Child Health, University of Nottingham, Queens Medical Centre, Nottingham NG7 2UH, UK

**Keywords:** prostate cancer, hand pattern, case–control study

## Abstract

**Background:**

The ratio of digit lengths is fixed *in utero*, and may be a proxy indicator for prenatal testosterone levels.

**Methods:**

We analysed the right-hand pattern and prostate cancer risk in 1524 prostate cancer cases and 3044 population-based controls.

**Results:**

Compared with index finger shorter than ring finger (low 2D : 4D), men with index finger longer than ring finger (high 2D : 4D) showed a negative association, suggesting a protective effect with a 33% risk reduction (odds ratio (OR) 0.67, 95% confidence interval (CI) 0.57–0.80). Risk reduction was even greater (87%) in age group <60 (OR 0.13, 95% CI 0.09–0.21).

**Conclusion:**

Pattern of finger lengths may be a simple marker of prostate cancer risk, with length of 2D greater than 4D suggestive of lower risk.

Prostate cancer is the most common non-skin male cancer in the UK (Cancer Research UK, 2010), but current knowledge of its aetiology is limited (Li *et al*, 2004). The ratio of 2nd and 4th digit length is fixed *in utero* (2D : 4D ratio), and is sexually dimorphic, lower in men than in women (Lutchmaya *et al*, 2004; Voracek *et al*, 2007). To date, only one longitudinal study has investigated digit ratio and prostate volume, PSA level and the prostate cancer risk (Jung *et al*, 2010). The ratio (2D : 4D) is negatively related to testosterone and related phenotypes, such as sperm counts, and positively related to oestrogen concentrations (Manning and Bundred, 2000). Accordingly, digit length pattern may act as a proxy indicator for the underlying prenatal testosterone levels. We therefore investigated this in a large case–control study of prostate cancer to explore whether there is any association between hand pattern and prostate cancer risk.

## Materials and methods

Information was collected on 1524 non-screen-detected prostate cancer cases and 3044 community-based controls during 1994–2009, with eligible cases being men aged ⩽80 years, currently living in the UK. Prostate cancer cases were identified from three large hospitals, two of them within Trent region in the UK, including Nottingham City Hospital and The Royal Hallamshire Hospitals in Sheffield together with The Royal Marsden NHS Foundation Trust Hospitals, London and Surrey. Controls were recruited via general practitioners of cases who were free of urinary tract symptoms as identified by an International Prostate Symptom Score questionnaire (score of ⩽7 out of 35). A postal questionnaire was sent to all eligible participants to collect information on selected exposures including right-hand pattern. Subjects were asked to identify the finger length pattern on their right hand as nearest to series of pictures, with clear instruction of how best to compare their hand with the pictures provided. There were three illustrations indicating: the index finger longer than the ring finger, the index equally as long as the ring finger and the index shorter than the ring finger. The latter was used as the reference category. The study received ethical committee approval (MREC/99/4/013, 07/MRE04/29).

### Statistical analysis

Unconditional logistic regression (SPSS version 16) was used to generate odds ratios (ORs) and 95% confidence intervals (CIs). To control for confounding, age and social class were added to the model; age was included as a continuous variable, whereas social class was fitted as a categorical variable.

## Results

Response rates to the questionnaire for cases and controls were 83 and 70%, respectively. The general characteristics of the study population are shown in Table 1. The median age of advanced cases is higher than that of the controls (62 compared with 57). Subjects with past smoking history appeared to show a small increase in risk compared with non-smokers (OR 1.20, 95% CI 1.03–1.39). Social class, education and marital status were not associated with prostate cancer risk (all CIs include 1). More than 90% of subjects were Caucasian.

Table 2 shows risk estimates for fingers reported to be of approximately equal length and index finger longer than ring finger length and as compared with index shorter than ring finger pattern; the latter reported pattern showing a statistically significant decreased prostate cancer risk with an OR of 0.67; 95% CI 0.57–0.80.

## Discussion

The study was a large case–control study with data collected over a period of 15 years, with similar rates of cases and controls recruitment. The subjects were asked to self-identify their pattern of index (2D) as compared to ring finger (4D). The results showed a negative association between length of 2D greater than 4D and prostate cancer risk (OR 0.67, 95% CI 0.57–0.80) in all ages and at ages <60 (OR 0.13, 95% CI 0.09–0.21) (results not shown). These negative associations suspect that lower prenatal activity of testosterone is protective against prostate cancer later in life.

The only study to investigate the relationship between digit length pattern and prostate cancer is the Korean Cohort study (366 subjects), which found a significant negative association between digit ratio and PSA (*r*=−0140, *P*=0.007) (Jung *et al*, 2010). Those with lower digit ratio had higher mean PSA level and higher risk of prostate biopsy (OR 1.75, 95% CI 1.07–2.84) and prostate cancer (OR 3.22, 95% CI 1.33–7.78).

Pictures of the right hand were provided to aid the response as there is a greater sex difference in 2D : 4D on the right hand than on the left hand (Williams *et al*, 2000). The procedure was particularly successful in terms of response rate (99% of eligible subjects responded to the question).

It has been suggested that intrauterine exposure of hormones influences the development of other adult-onset diseases (Manning and Bundred, 2000), including a large study on finger pattern and osteoarthritis risk, in which lower digit ratio was associated with osteoarthritis (Zhang *et al*, 2008). In the latter study, digit lengths were physically measured on hand radiographs using vernier callipers to achieve a high degree of accuracy and repeatability. This was considered impractical and unethical for our study; hence, hand radiographs were not used. Instead, we used a simpler way to identify the pattern of 2nd and 4th finger by self-reported comparison of the hand with pictures. The self-reported finger length, however, raises the possibility of measurement error, as discussed by Caswell and Manning. In their study, they used two different approaches to measure 2D : 4D, including finger length measured from photocopies of the ventral surface of hands (photo 2D : 4D) and self-reported finger length measured directly from the finger (S–R 2D : 4D). The results suggested that self-reported 2D : 4D showed some more extreme values when compared with photo 2D : 4D. It was concluded, however, that a large sample size would reduce the effect size of this (Caswell and Manning, 2009); hence, this possible error is unlikely to have a large effect in our study.

The finger length relationship seen in our study is also in keeping with equivalent studies in breast cancer risk based on current understanding of the role of hormonal patterns *in utero*. Women with a high ratio of 2D : 4D (indicative of higher prenatal oestrogen exposure) are at greater risk of breast cancer. Women with the more ‘feminine’ pattern of digit length (2D : 4D high – ring finger closer in length or shorter than the index finger) were also more likely to present at a younger age (Manning and Bundred, 2000).

Although finger length in humans has been studied for decades, its relationship with hormones has been determined only by one relatively small-scale study (Lutchmaya *et al*, 2004). In humans, the growth and pattern of digits and the differentiation of gonads is controlled by the homeobox genes *HOXA* and *HOXD*. Therefore, gonadal foetal products such as testosterone may influence finger morphology (Manning *et al*, 2003). For example, a high concentration of testosterone, indicating high prenatal testicular activity leads to low 2D : 4D ratio. The negative correlation between digit ratio and hormone profile has been used as a marker to predict offspring sex ratio and sporting ability (Robinson and Manning, 2000; Williams *et al*, 2000; Manning and Taylor, 2001; Manning *et al*, 2002). The ratio (2D : 4D) is greater in the right hand than in the left hand, and has a higher sensitivity with foetal androgens than the left hand (Williams *et al*, 2000). A high 2D : 4D ratio in male right hands was associated with germ cell failure due to azoospermia or oligospermia with no motility; furthermore, testosterone assays from 58 male subjects were negatively associated with 2D : 4D ratio in the right hand (*P*=0.03), which was not seen in the left hand (Manning *et al*, 2000). Twin studies suggest that there is also a possible genetic role in addition to any prenatal environmental influence on this hormonally related skeletal ratio in both men and women (Paul *et al*, 2006; Gobrogge *et al*, 2008).

A protective effect of a high 2D : 4D hand pattern on prostate cancer risk was observed. High 2D : 4D hand pattern may be the marker of low prenatal androgenic activity, suggesting the importance of hormone modulation in *utero* on prostate cancer risk. Hand pattern might represent a simple marker for prostate cancer risk, particularly in men age under 60 years.

## Figures and Tables

**Table 1:** General characteristics of study population

**Characteristics**	**Advanced cases (*n*=1524)**	**Controls (*n*=3044)**	**OR (95% CI lower–upper)^†^**
Median age	62 (36–80)	57 (30–80)	1.09 (1.08–1.10)
			
*Smoking*			
Never	500 (32.6)	1104 (37.5)	1.00
Ex-smokers	819 (53.4)	1359 (44.7)	1.20 (1.03–1.39)
Smokers	215 (14.0)	543 (17.9)	1.03 (0.84–1.27)
			
*Social class* a			
I–II	704 (47.3)	1442 (48.7)	1.00
IIIN–IIIM	658 (44.2)	1319 (44.5)	1.07 (0.94–1.23)
IV–V	126 (8.5)	200 (6.8)	1.15 (0.89–1.50)
			
*Education*			
None	549 (36.6)	708 (25.2)	1.00
GCSE	246 (16.4)	552 (19.6)	0.81 (0.66–1.00)
A level	91 (6.1)	203 (7.2)	0.75 (0.55–1.01)
Higher degree	614 (40.9)	1349 (48.0)	0.77 (0.64–0.92)
			
*Marital status*			
Married	1246 (81.8)	2585 (84.9)	1.00
Ex-married	207 (13.6)	329 (10.8)	1.19 (0.97–1.46)
Single	71 (4.7)	129 (4.2)	1.31 (0.95–1.81)
			
*Ethnicity*			
White	1466 (96.4)	2954 (96.9)	1.00
Black	29 (1.9)	33 (1.1)	2.40 (1.38–4.15)
Asian	9 (0.6)	40 (1.3)	0.59 (0.27–1.25)
Others	16 (1.1)	20 (0.7)	1.91 (0.91–3.97)

Abbreviations: CI=confidence interval; GCSE=General Certificate of Secondary Education; OR=odds ratio.

^†^Adjusted for age.

a Categories of social class were made by identifying longest held job by participants.

**Table 2:** Right-hand pattern and prostate cancer risk

**Finger pattern**	**Advanced cases (%)**	**Controls (%)**	**OR^a^**	**95% CI lower–upper**	***P*-value**
Index shorter than ring	872 (57.2)	1570 (51.6)	1.00		
Index equal to ring	305 (20.0)	538 (17.7)	1.05	0.88–1.25	0.580
Index longer than ring	347 (22.8)	936 (30.8)	0.67	0.57–0.80	<0.001
Total	1524 (100.0)	3044 (100.0)			

Abbreviations: CI=confidence interval; OR=odds ratio.

a Adjusted for age and social class.
